# Optical transparent metamaterial emitter with multiband compatible camouflage based on femtosecond laser processing

**DOI:** 10.1515/nanoph-2024-0763

**Published:** 2025-03-18

**Authors:** Shu-Wen Zheng, Xiu-Yu Chen, Jin-Long Huang, Kun Yu, Meng-Dan Qian, Yu-Fang Liu

**Affiliations:** Hennan Key Laboratory of Infrared Spectrum Measures and Applications, School of Physics, 12568 Henan Normal University, Xinxiang, Henan 453007, China; Institute of Physics, Henan Academy of Sciences, Zhengzhou, 450046, China

**Keywords:** IR camouflage, metamaterial, femtosecond laser direct writing, thermal management, spectral selectivity

## Abstract

Infrared (IR) camouflage has garnered growing attention with progress in IR detection technology. The emergence of metamaterial with powerful electromagnetic field regulation ability provides an effective solution for thermal emission manipulation in IR camouflage. However, the intricated micro/nano machining technology of metamaterial greatly limits its moving toward practical application, and single-band IR camouflage makes it difficult to resist multiband cooperative detection systems. Here, a flexible, fine, and mask-free femtosecond laser direct writing (FsLDW) technology was introduced to pattern on ultra-thin metals. Based on this efficient technique, the optically transparent metamaterial emitter with multiband compatible camouflage is fabricated. The emitter is demonstrated to achieve high reflectance (*R*_3–5 µm_ = 0.79 and *R*_8–14 µm_ = 0.70) in the dual-band atmospheric window and low reflectance (*R*_1.06 µm_ = 0.3, *R*_1.55 µm_ = 0.1) for IR and laser stealth. In addition, the high emissivity (*ɛ*_5–8 µm_ = 0.64) for the nonatmospheric window effectively dissipates the accumulated heat, showing promising prospects in radiative cooling by comparison with Ag at the same heating power. This work offers a clue for coordinated control of multiband electromagnetic waves and heat through simple structural design, which is expected to promote its camouflage applications and thermal management in the military.

## 1 Introduction

Camouflage technology involves achieving low observability across various spectral range, e.g., visible (VIS) [1], [2], [3], [4], [5], infrared (IR) [6], [7], [8], [9], or radar spectra [10], [11], [12], [13], which is significant in modern military field. In particular, IR camouflage has garnered growing attention due to the prevalent use of homing systems that depend on IR signals for tracking targets. The Steffen–Boltzmann law points out that the intensity of the IR signal emitted by an object is proportional to the fourth power of the absolute temperature (*T*) and the object’s surface emissivity (*ɛ*) [14], [15], [16], [17]. The temperature regulation for realizing IR camouflage often introduces additional heat instability [18], [19], [20], [21]. By contrast, controlling the surface emissivity offers a more flexible and efficient approach [17], [22], [23], [24], [25], [26], [27], [28], [29], [30]. The traditional infrared selective emitter (ISE), which is made of metal, dielectric, has low emissivity in the atmospheric window and high emissivity in the nonatmospheric window. However, most of these structures are opaque, which hinders their application in specific scenarios, such as cockpit window, canopies, and lampshades. Besides, single-band IR camouflage has been difficult to meet the diversity of modern detection technologies.

The emergence of metamaterial presents new possibilities in the realm of multiband emissivity modulation, enabling precise manipulation of electromagnetic behavior via various microstructural designs [13], [25], [31], [32], [33]. To date, photolithography technique, electron-beam lithography, focused ion beam etching, and inductively coupled plasma etching have demonstrated substantial potential in the processing of metal micro–nanostructures [13], [25], [27], [34], [35], [36], [37]. Despite significant research advancements of preparation of metamaterials, there are still some inevitable problems with these technologies, such as low precision, poor stability, and complicated process. Recently, femtosecond laser direct writing (FsLDW) technology has emerged as a remarkable approach for its high spatial resolution, arbitrary shape designability, and mask-free [38], [39]. Liu et al. proposed a method based on FsLDW technology to achieve high solar energy absorption on the surfaces of different metallic materials [40]. Chen et al. fabricate an all-dielectric metasurface absorber using FsLDW technology, which displays significant improvement of absorption in the infrared region of 8–14 μm [41]. In contrast, FsLDW utilizes direct laser processing, enabling the creation of highly precise 3D microstructures, which can modulate infrared emissivity and reflectivity, providing enhanced thermal management in camouflage applications. As a powerful micro–nanofabrication technique, FsLDW enables the creation of highly precise 3D microstructures, which can modulate infrared emissivity and reflectivity, providing enhanced thermal management in camouflage applications [42]. FsLDW technology provides a current solution for the compatible regulation of radiation spectrum in wide band, which is great important to improve the combat performance of camouflage technology in modern complex battlefield.

In this work, a flexible, fine, and mask-free FsLDW technology was introduced to pattern on ultra-thin metals. Based on this efficient technique, the optically transparent metamaterial emitter with multiband compatible camouflage is fabricated. It consists of ultra-thin metal Ag, dielectric material ZnS, and reflection layer ITO. The on-top metal is patterned with a resolution of approximate 2 μm using FsLDW technology [43]. This metamaterial emitter presents several advantageous characteristics: (1) the optically transparent emitter exhibits low emission at atmospheric windows (*ɛ*_3–5 µm_ = 0.21, *ɛ*_8–14 µm_ = 0.30) for IR camouflage and high emission (*ɛ*_5–8 µm_ = 0.64) at nonatmospheric window for radiative cooling. The actual temperature (216.3 °C) of the sample could be 26 °C lower than that of the traditional metal material (242.3 °C) under the same heating power conditions (65W), indicating the excellent thermal management capability. (2) It demonstrates effective laser camouflage capabilities by low reflectance at wavelengths of 1.06 μm and 1.55 μm. (3) The absorption performance of this emitter is almost not affected by angle within the large angle range, which shows that the absorption peak is maintained at 80 % with the angle range of 0∼70°. (4) The metamaterial emitter can maintain excellent performance at temperature up to 300 °C.

## 2 Results and discussions

**Figure 1:**
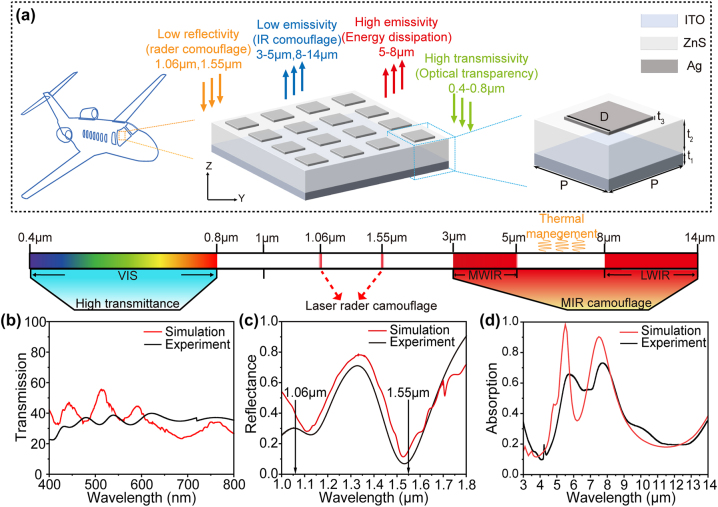
Optical transparent metamaterial for multispectral camouflage. (a) Schematic of the multifunctional optically transparent multiband emitter. (b) The simulated and experimentally measured VIS transmittance of the emitter. (c) A side-by-side analysis of simulated and measured reflectivity spectra, illustrating the emitter’s spectral response across various wavelengths. (d) The simulated and experimentally emissivity of the designed nanostructures in 3–14 µm.

Multispectral camouflage necessitates adherence to distinct wavelength band detection principles. Specifically, within the VIS spectrum region, maximizing transmittance ensures seamless blending with the surroundings. In the IR atmospheric window, minimizing emittance is crucial to evade detection by infrared detection. Conversely, in the nonatmospheric window (5–8 μm), enhancing emittance facilitates radiative cooling. Additionally, for laser wavelengths commonly employed in light detection and ranging (liDAR) systems, such as 1.06 μm and 1.55 μm, reducing the laser echo energy is imperative [44]. In order to achieve all the requirements mentioned above, we prepared a metamaterial emitter from the material selection and structural design shown in Figure 1(a), which consists of grating array (Ag), dielectric spacer layer (ZnS), and antireflective layer (ITO) from top to bottom, and the thickness is 20 nm, 650 nm, and 200 nm, respectively. Within each periodic cell of the structure, the top Ag grating has a width (*D*) of 3 μm, while the emitter period (*P*) is set to 5.4 μm. Ag is selected due to its high IR reflectivity across a broad range [45], [46], and its grating array is utilized as a metallic layer to enhance electromagnetic resonance. ITO not only has high transmittance in the VIS but also has high reflectance in the infrared band. The dielectric layer ZnS is an infrared-transmitting dielectric material with a broad transmission band ranging from 0.3 to 14 μm, which is widely used in military applications. In addition to this, ZnS possesses a high refractive index (*n* = 2.2–2.3), which facilitates the reduction of spacer thickness [47].

Across the VIS spectrum ranging from 400–760 nm, both the ITO film and ZnS film exhibit high transmittance. Figure 1(b) presents the simulated and experimental VIS transmittance of the emitter. Specifically, the average transmittance is 35.5 % in simulation and 34.9 % in experimentation, respectively. Regarding the liDAR’s laser wavelength, the incorporation of a grating structure effectively enhances laser energy dissipation, facilitating simultaneous low reflectance at both 1.06 μm and 1.55 μm. As illustrated in Figure 1(c), the reflectance at 1.06 μm and 1.55 μm wavelengths are below 0.3 and 0.1, respectively, showing great liDAR camouflage performance. Turning to IR camouflage, the three-layer metal–dielectric–metal (MDM) structure can site-selectively absorb/emit electromagnetic wave in the nonatmospheric window. The absorption spectra of the emitter within the range of 3–14 μm are depicted in Figure 1(d), both simulated and measured. From Figure 1(d), it can be observed that the absorption spectrum displays two distinct absorption peaks at wavelengths of 5.5 μm and 7.6 μm. The average emissivities in the atmospheric windows of 3–5 μm and 8–14 μm are measured to be 0.21 and 0.30 (the simulated values: 0.31 and 0.30), respectively, indicate favorable IR camouflage characteristics. Moreover, with an average emissivity of 0.64 in the nonatmospheric window range of 5–8 μm (the simulated value: 0.68), efficient dissipation can be achieved to maintain thermal stability for the operation of the emitter.

**Figure 2:**
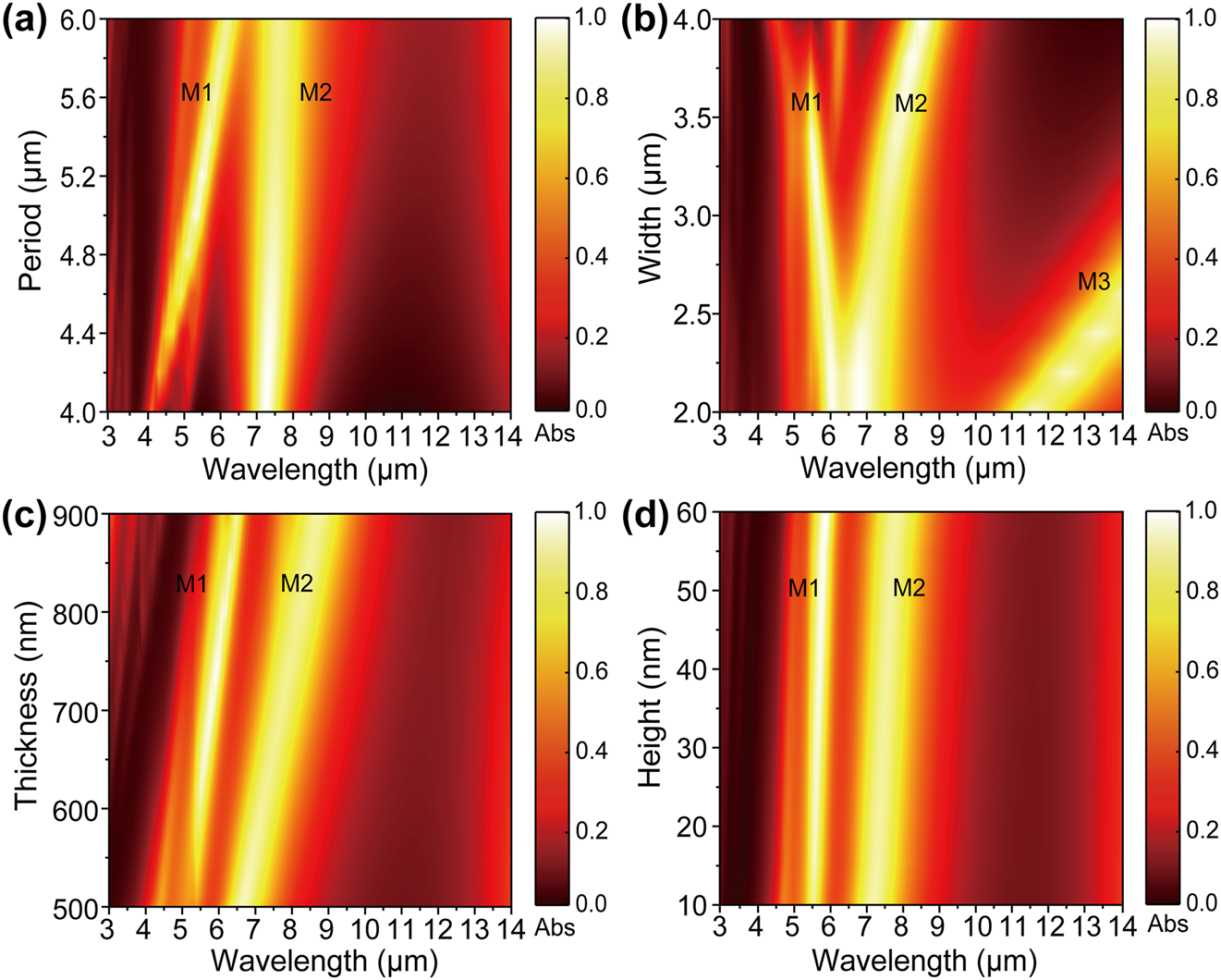
Calculated results for metamaterial in infrared wavelength. (a) The absorption of the emitter varied with a changed period (*P*). (b) The absorption of the emitter varied with a changed grating with (*D*). (c) The absorption of the emitter varied with a changed dielectric layer thickness (*t*_2_). (d) The absorption of the emitter varied with a changed grating height (*t*_3_).

The absorption characteristics of the metamaterial are closely related to its geometric structure. To investigate the influence of structural parameters on emitter performance, we systematically varied the period, grating width, dielectric layer thickness, and grating height. The simulated absorption spectra of the emitter, corresponding to various geometric parameters, are illustrated in Figure 2(a)–(d). Figure 2(a) shows the IR absorption spectra at different periods, the M1 shifts to a long wavelength with an increase of period, but the M2 barely changes. Notably, there is a direct proportionality between the resonant peak of M1 and the period. As seen in Figure 2(b), the resonance band located at the shorter wavelength region (∼6.5 µm) as a hybrid resonance when *D* is less than 2.5 µm, while it splits into two peaks (M1 and M2) after *D* is over 2.5 µm. In contrast, the absorption peak (M3) shifts to a long wavelength with the increase in width of the grating. This aligns with the expectations of the inductance-capacitance (LC) model, which indicates that the resonant center wavelength is directly related to the width of the grating (MATLAB simulations were conducted to calculate the resonance peaks M3 for different grating widths is shown in Figure S1). As the thickness of the dielectric layer increases from 500 to 900 nm, the resonant wavelength at M1 and M2 show a redshift along with the enhanced absorbance intensity in Figure 2(c). In addition, the variations in the height of the metal grating have minimal effect on the absorption spectra shown in Figure 2(d).

**Figure 3:**
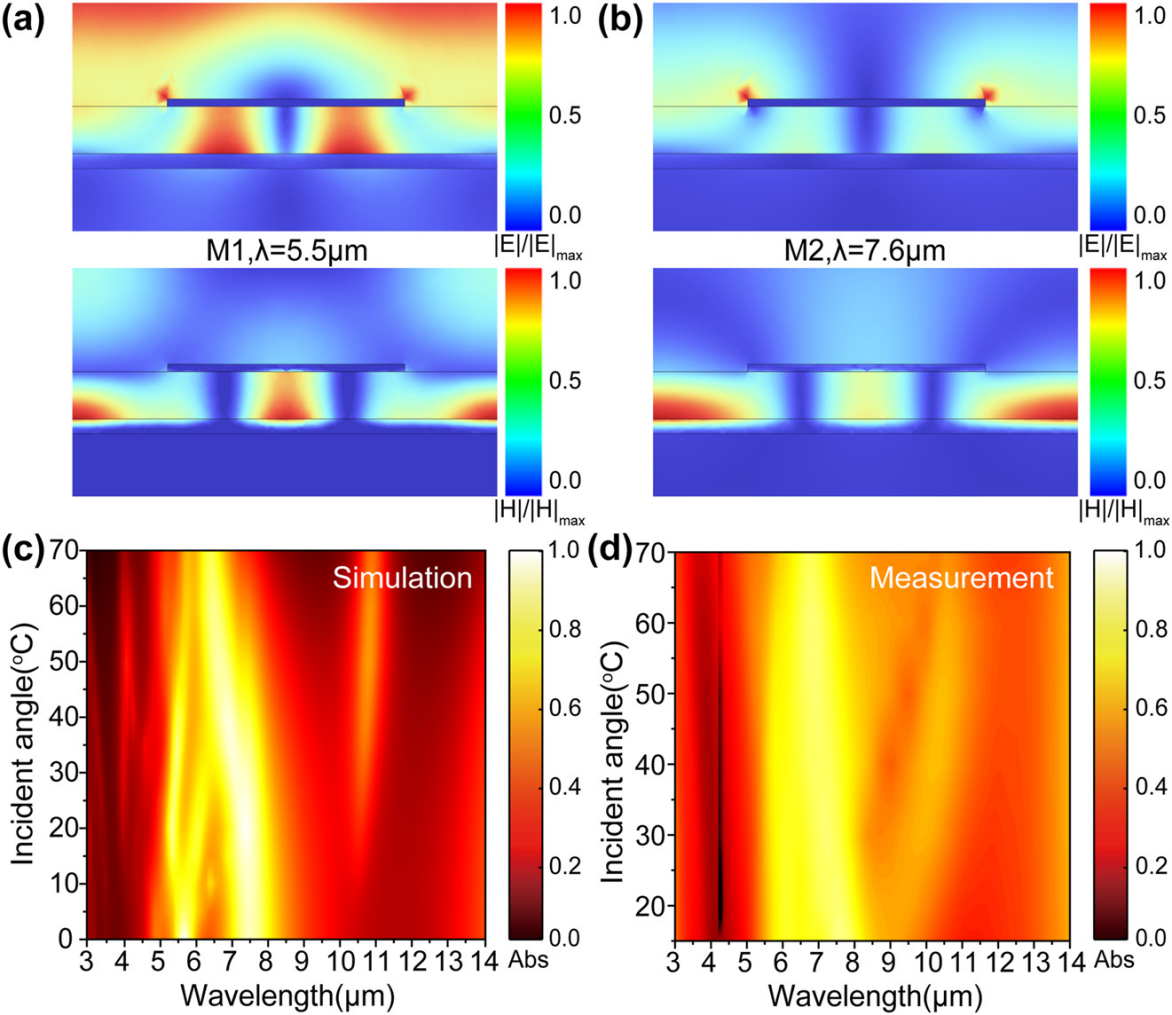
The physical mechanism and angular sensitivity of the structure. (a, b) Simulated electric field (the top row) and magnetic field intensity/(the bottom row) at resonance wavelengths of 5.5 μm and 7.6 μm, respectively. (c, d) Simulated and experimental absorption spectra for incident angles from 0° to 70°, respectively.

Then, to further verify the optical properties of the emitter, simulations were conducted to analyze the distributions of electric and magnetic fields at the resonant wavelengths M1 [depicted in Figure 3(a)] and M2 [depicted in Figure 3(b)]. The field distributions confirm that the absorption properties of the structure are attributed to the electric and magnetic resonances excited within the tri-layer configuration. It is noteworthy that the field distributions at M1 and M2 exhibit certain similarities. As shown in Figure 3(a) and (b), the electric field distribution at the M1 and M2 exhibits remarkable surface plasmon polaritons (SPP) resonance characteristics. Specifically, the electric field is mainly concentrated near the interface of metal and medium, indicating the strong excitation of SPP waves at this interface. The SPP arises from the interaction between free electrons on the metal surface and electromagnetic waves. Besides, the magnetic field distributions at the M1 and M2 confined within the intermediate ZnS film are a typical indication of the third-order magnetic resonance, while that at the interface between the ZnS film and the underlying layer are a sign of the antireflection resonance. (For details, see Supporting Information S2.) The distinct lies in the fact that, at M1, the influence of higher-order magnetic resonance is more pronounced, whereas at M2, the impact of enhanced reflection resonance is more notable.

The angular dependence of the emitter is validated by simulating and measuring the absorption spectra at various incidence angles as shown in Figure 3(c) and (d). The absorption spectrum exhibited minimal variation at the incidence angle below 40°, showing angle-independent absorption for transverse electromagnetic (TEM) modes due to the high symmetry of the structure. As the incidence angle increased beyond 40°, a resonant absorption was observed at a wavelength of approximately 10.6 μm, while the maximum peak value is lower than 0.5, as shown in Figure 3(d). Both the simulated results and the measured results demonstrated that the metamaterial has great angle-independent behavior in a wide-angle range.

**Figure 4:**
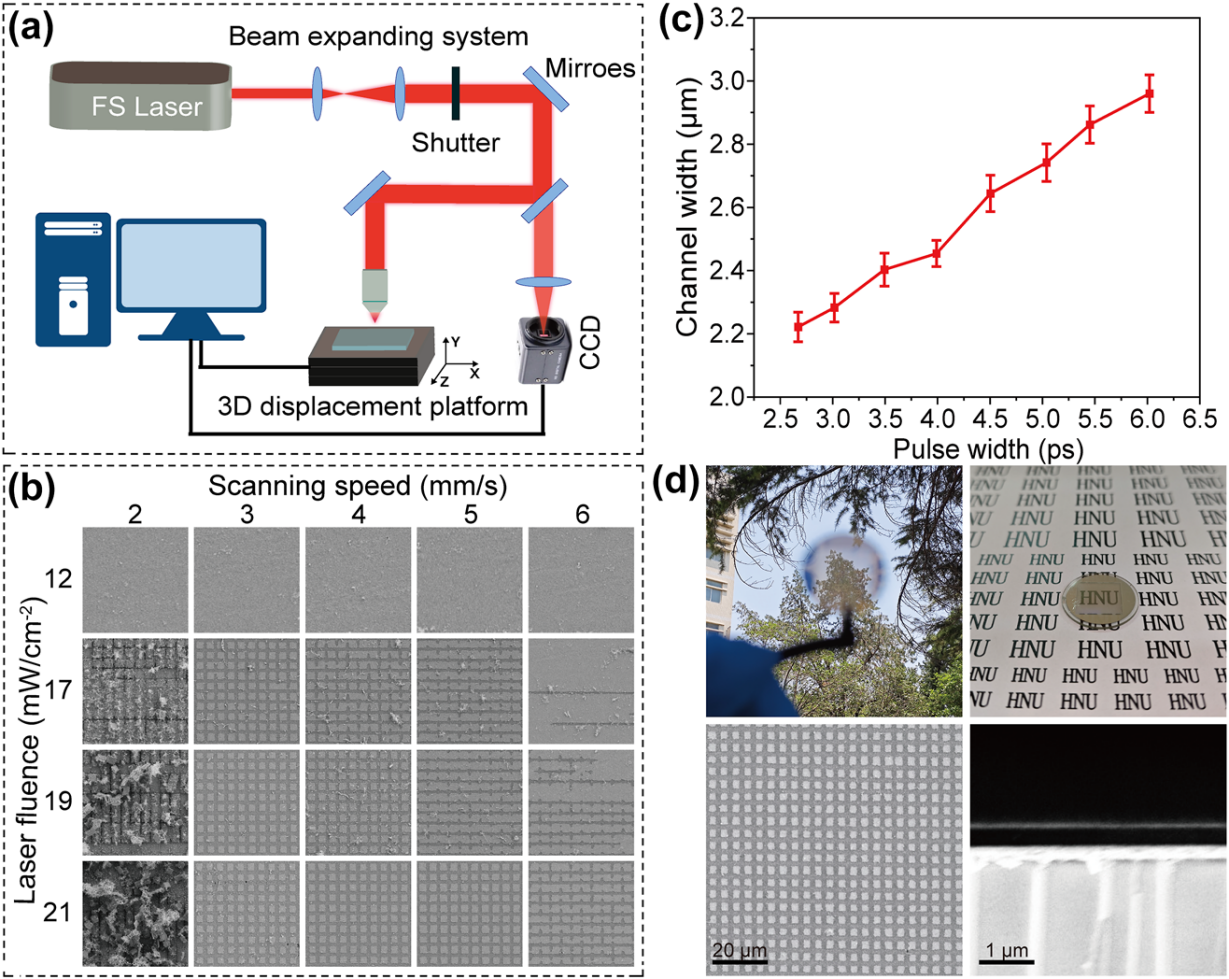
The samples are processed via femtosecond laser direct technology. (a) Femtosecond laser micro/nanofabrication system. (b) SEM images of a metasurface sample created using a femtosecond laser, captured at various scan speeds and laser fluences. (c) Variation of channel width of metallic Ag as a function of pulse width. (d) VIS image, top view and cutaway SEM image of the fabricated sample.

The emitter was fabricated through electron beam evaporation and FsLDW technology. A glass substrate served as the base layer, upon which the bottom ITO layer, ZnS layer, and the top Ag layer were sequentially deposited via electron beam evaporation. Subsequently, an attempt was created a one-dimensional (1D) metal grating on the top Ag layer utilizing the FsLDW process. Nevertheless, the optical performance of the resulting one-dimensional grating structural was found to be unsatisfactory. Therefore, it was decided to switch to a two-dimensional (2D) grating design, which promised superior optical performance and structural stability. (The fabrication of the one-dimensional grating through FsLDW is depicted in Figure S3.) The experimental setup for the femtosecond laser micro/nanofabrication system is illustrated in Figure 4(a). A femtosecond laser beam with a wavelength of 1,030 nm is focused on the Ag film for patterning. Upon the interaction of the femtosecond laser pulse with the metal surface, the electrons inside the metal absorb light energy over a brief period, causing a rapid elevation in metal temperature. These stimulated electrons subsequently interacted with the phonons within the metal, transferring their power to the lattice. As the lattice temperature gradually increases to a critical threshold, the metal is melted and ablated [48], [49], [50]. Before the patterning process, systematic experiments were performed to assess the effects of laser power density (12, 17, 19, and 21 mW/cm^2^) and scanning speeds (2, 3, 4, 5, and 6 mm/s) on the ablation and removal of the Ag film. Figure 4(b) shows the scanning electron microscope (SEM) images of the sintered regions on Ag film under various processing conditions. With the increase of laser fluence, the ablation area will gradually increase. The optimal removal was observed at a moderate scanning speed. The excessive scanning speeds led to insufficient energy absorption by the electrons in the metal within a short period, thereby preventing the effective removal of the metal material. Conversely, excessively low scanning speed results in energy accumulation and the metal being ablated away, affecting the next layer of material. After conducting a comparative analysis, we determined that the most suitable femtosecond laser scanning speed was 4 mm/s with a laser fluence of 21 mW/cm^−2^. We next explored the effect of laser pulse widths on metal Ag film. As depicted in Figure 4(c), the line width of the sintered area gradually broadens with an increase in the laser pulse width. Figure S4 shows the SEM images of the sintered region on Ag films under different laser pulse widths. It can be seen that the whole patterned Ag film has uniform structure and defined profiles. Therefore, the FsLDW system can achieve flexible machining of the metal surface structure without a mask by simply controlling the parameters of the laser pulse incident on the metal. By fine-tuning these parameters, the amount of residual material sputtered back onto the surface has been effectively reduced. In addition, we used an ear cleaning bulb to gently blow off any residual material from the surface after the laser processing, ensuring a cleaner surface for further analysis. Figure 4(d) shows photographs of the fabricated sample and the cross-sectional SEM image. The transparency of the emitter is confirmed by the image captured through the window, demonstrating its high transparency. As illustrated in Figure 4(d), the metal pattern is homogenous squares with an ablated square width of 3 µm. The thicknesses of Ag grating, ZnS, and ITO layers are approximately 18 nm, 650 nm, and 200 nm respectively, which is consistent with the parameters derived from the optimized simulations.

**Figure 5:**
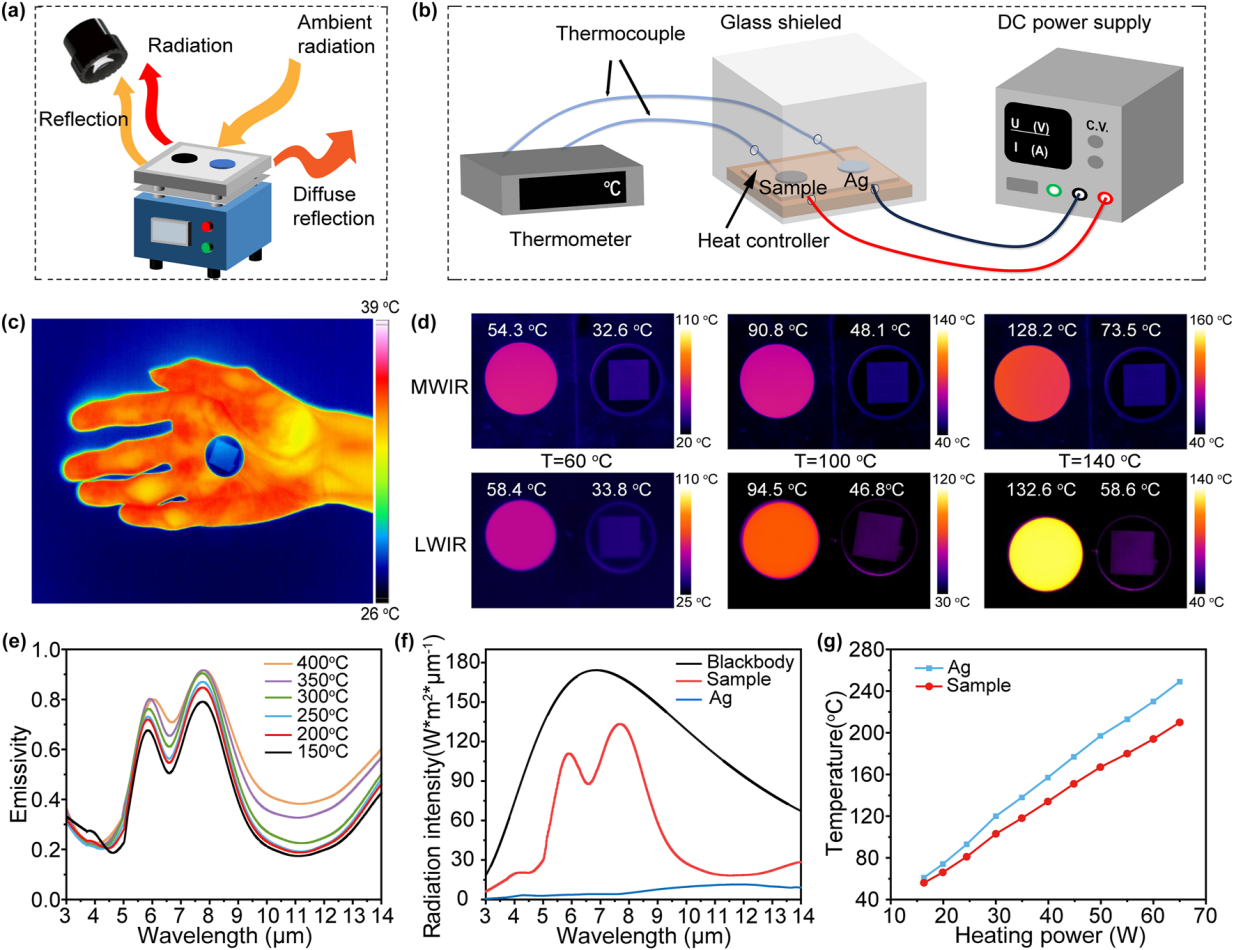
Experimental performance of IR camouflage and thermal management. (a) Thermal IR imaging experimental setup: (working band: 3 μm–5 μm and 7.5 μm–14 μm). The IR emissions emanating from the sample consist of both the sample’s own radiation and reflected ambient radiation. (b) Thermal management measurement setup. (c) The sample’s ability to camouflage within IR bands at ambient conditions. (d) Thermal imaging & radiation temperatures under varying background temperatures of samples and reference object in 3–5 µm (the top row) and 7.5–14 µm (the bottom row). (e) The spectral emissivity of the emitter is measured across different temperatures utilizing Fourier transform infrared (FTIR) spectroscopy. (f) The spectral radiation profiles of the sample, a Ag film, and a blackbody at various wavelengths. (g) The actual temperatures on the surface of both the sample and the Ag film.

To validate the IR camouflage and thermal management characteristics of the emitter, we employed the testing systems illustrated in Figure 5(a) and (b), respectively. Specifically, as shown in Figure 5(a), the sample and the glass wafer of the same dimensions were heated simultaneously on the heating table, and IR images of both the sample and the glass wafer placed on a metal substrate (*ε* = 0.2) were captured by the IR camera. It is imperative to consider sunlight reflection in the design of IR camouflage materials, as the detection capability of a thermal imager encompasses not only the radiated power of the sample but also the ambient radiation power reflected by it. Consequently, when calculating the radiation temperature in various states, it is necessary to incorporate an additional component representing the radiation temperature reflected from the surrounding environment (Supporting Information S5). In order to achieve effective IR camouflage, objects need to have a similar radiation temperature as the background, making them nearly “invisible” to IR cameras. Figure 5(c) showcases that, under natural conditions and observed through an IR camera, the radiation temperature of the sample is about 27 °C, which is lower than that of the human body (about 35 °C). Its radiation signature is similar to the surrounding environment, ensuring that the sample is virtually undetectable by IR cameras at room temperature. Figure 5(d) presents the samples and the glass wafer in (mid-wave infrared) MWIR (the up row) and (long-wave infrared) LWIR (the bottom row) images across a temperature spectrum ranging from 60 °C to 140 °C. As the heating temperature gradually escalates, both the sample and the glass wafer exhibit a corresponding increase in radiative temperature, as corroborated by the accompanying Figure S6. Notably, the sample consistently maintains a lower radiation temperature compared to the glass wafer. Specifically, at a heating temperature of 140 °C, the glass substrate attains 128.2 °C (132.6 °C) in MWIR (LWIR), whereas the sample remains at a lower temperature of 70.5 °C (64.2 °C). Apparently, the radiation temperatures of our fabricated samples are closer to that of the background in both detected bands, representing that the proposed emitter presents an outstanding performance in suppressing thermal radiation compared with glass and significantly diminishing the chances of detection in IR camouflage applications. Furthermore, the thermal stability of the prepared sample was examined by assessing its spectral emissivity at varying temperatures. The emissivity of the emitter was measured in the actual thermal environment spanning 150–300 °C, and the results are presented in Figure 4(e). The sample effectively maintain its spectral selectivity well all over the measured temperature range and the average emissivity data of the emitters over the 3–14 µm range undergoes neglectable variation, making it a suitable choice for a wide array of application environments with varying thermal demands. In order to quantify the average emissivity at a given *T* within a specific wavelength band, one can utilize the relationship between spectral emissivity and blackbody irradiation, as demonstrated in the following equations [46]:

(1) $$\bar{\varepsilon }=\frac{\overset{\lambda_{2}}{\underset{\lambda_{1}}{\int }}\varepsilon_{\lambda }E_{b\lambda }\left(T\right)d\lambda }{\overset{\lambda_{2}}{\underset{\lambda_{1}}{\int }}E_{b\lambda }\left(T\right)d\lambda }$$

(2) $$E_{b\lambda }=\frac{2\pi hc^{2}}{\lambda^{5}\exp\left(hc/k\lambda T-1\right)}$$

In which the spectral irradiance of the blackbody, wavelength, and temperature are represented by *E*_*bλ*_, *λ*, and *T*, respectively. The parameters *h*, *k*, and *c* represent Planck’s constant, Boltzmann’s constant, and the speed of light in vacuum. As calculated by equations (1) and (2), the designed structure exhibits remarkable spectral selectivity, as evidenced by its low average emissivities in the MWIR (*ɛ*_3–5 µm_ = 0.21) and LWIR (*ɛ*_8–14 µm_ = 0.30) regions at 150 °C, while achieving a substantial average emissivity of 0.64 within the 5–8 µm band (the average emissivity of the samples in different bands in the temperature range of 150–400 °C is shown in Figure S7 of Supporting Information). Therefore, the prepared sample is applicable not only for IR camouflage but also for thermal management owing to its powerful radiative ability at nonatmospheric wavelengths. In addition, the performance of our work is compared with published optically transparent multiband compatible camouflage as shown in S8. To evaluate the thermal management performance of the emitter, compassion of spectral radiation between the sample and an Ag film of equal size is analyzed. Figure 5(f) illustrates the spectral radiation of blackbody, the sample, and the Ag film at a temperature of 150 °C. Within the atmospheric transparency windows (3–5 µm and 8–14 µm), the radiative power of the blackbody is 149.13 W/m^2^ and 676.44 W/m^2^ of radiant power, respectively. In contrast, the sample demonstrates a substantial attenuation, with radiative powers of only 31.21 W/m^2^ and 206.93 W/m^2^ within these bands. Notably, the Ag film exhibits minimal radiated power due to its intrinsically low emissivity. These findings underscore the sample’s efficacy in reducing IR signatures across key wavelength ranges. Especially within the 5–8 µm band, the sample dissipates a power of 304.03 W/m^2^, which is 28 times that of Ag film.

To further quantify the thermal management capability of the sample, a comparative analysis of the sample and Ag film’s real-time temperature responses under varying ambient temperatures is presented. As shown in Figure 5(b), heat was introduced through a controlled power supply, ensuring a stable and adjustable thermal input, while the surface temperatures were accurately measured using a precision thermometer interfaced with a thermocouple. To mitigate disturbances from ambient airflow, a glass shield was implemented, effectively isolating the experimental setup and ensuring that temperature readings were uncompromised by external factors. Figure 5(g) illustrates that the sample’s temperature is consistently lower than that of the Ag film, under identical heating conditions. As the heating power intensifies, both materials undergo a commensurate increase in surface temperatures, while the disparity between their temperatures becomes increasingly pronounced. Specifically, at a heating power of 65W, the sample maintains an actual temperature of 216.3 °C, a remarkable 26 °C cooler than the Ag film’s 242.3 °C. This disparity stems from the sample’s enhanced radiative power output as its temperature rises. In contrast, the Ag film’s emissivity remains relatively stable, impeding to dissipate heat through radiation efficiently. The thermal management efficacy of the sample can be attributed to its superior band-selection property, facilitating the dissipation of accumulated heat through nonatmospheric windows. This will contribute to its outstanding thermal management performance at high temperatures.

## 3 Conclusions

In summary, we have fabricated an optically transparent emitter by FsLDW technique, which is proven to be a flexible processing technology without a mask by simple controlling the parameters of the laser pulse incident on the metal. The metamaterial emitter within the specific MDM structure with multiband compatible camouflage capability across VIS, laser, and IR bands incorporating thermal management. Our experimental outcomes underscore the emitter’s exceptional performance, featuring a high VIS transmittance, minimal reflectance of 10 % at laser wavelengths, low emissivity of 21 % and 30 % within the dual-band atmospheric window of IR detection, and a high emissivity of 64 % in the nonatmospheric window of 5∼8 µm. Furthermore, we have experimentally validated the thermal emissivity stability of this metamaterial emitter, revealing negligible variations in its emissivity spectrum across a broad temperature range from 150 °C to 300 °C, indicative of exceptional thermal stability. This work provides a new solution for coordinated control of multiband electromagnetic waves and heat through simple structural design.

## 4 Simulation and experimental section

Numerical simulations: The absorption performance of the emitter is evaluated through numerical simulation using COMSOL Multiphysics software. The addition of an ITO substrate significantly reduces light transmission. Because the substrate is sufficiently thick to block light transmission, transmission for the structure can be disregarded. Consequently, the absorption of the structure can be determined using the formula *A* = 1 − *R*.

Sample fabrication: In the fabrication of optically transparent multiband emitters, preparatory steps were undertaken. An ITO glass wafer, 1 mm in thickness, was first cleansed with acetone, followed by 15-min sonication process. This was further complemented by sequential ultrasonic treatments for 15 min each in ethanol and subsequently for 20 min in deionized water, effectively purging residual organic matter from the glass surface. Drying was accomplished through a gentle nitrogen stream. Electron beam evaporation is utilized to first apply the 650 nm ZnS layer, and then the 18 nm Ag layer is deposited.

Subsequently, the creation of intricately patterned Ag grating array on the top surface was accomplished via femtosecond laser (FemtoLY-20) processing. The sample was precisely positioned on a displacement platform, while a high-energy femtosecond laser beam – centered at 1,034 nm, with a pulse width of 380 fs and a repetition rate of 25 KHz – was employed. The laser’s output power was intricately modulated through program control software, synchronized with a Newport 3D displacement platform and shutter switch, to ensure precise pattern fabrication. The laser output power was adjusted by the program control software. Combined with the Newport 3D displacement platform and shutter switch, the pattern fabrication was conducted. This process culminated in the realization of the optically transparent multiband emitter.

Optical measurement: The emittance spectra of the sample for IR emissivity were acquired using a Fourier transform infrared (FTIR) spectrometer (Bruker 70 V). This spectrometer was equipped with a liquid-nitrogen-cooled mercury cadmium telluride (MCT) detector and a KBr beam splitter, ensuring exceptional sensitivity and accuracy. The standardized IR-563 blackbody served as the radiation source. During measurement, the samples were affixed to a temperature controller, directing their emitted power into the FTIR spectrometer. The spectral emissivity of the emitter was derived utilizing a well-established formula:

(3) $$\varepsilon \left(\lambda ,T\right)=\frac{M_{S}\left(\lambda ,T\right)-M_{am}\left(\lambda ,T\right)}{M_{b}\left(\lambda ,T\right)-M_{am}\left(\lambda ,T\right)}$$

Comparing the radiation intensities of the sample ( $M_{S}\left(\lambda ,T\right)$ ), blackbody ( $M_{b}\left(\lambda ,T\right)$ ), and background at a given *T* ( $M_{am}\left(\lambda ,T\right)$ ). Furthermore, to visualize the microscopic structure of the samples, scanning electron microscopy (SEM) images were acquired using a field-emission SEM (SUPRA40, Zeiss, Germany).

## Supplementary Data

Supplementary material associated with this article can be found in the online version.

## Supplementary Material

Supplementary Material Details
