# Supplementary material for: Optical transparent metamaterial emitter with multiband compatible camouflage based on femtosecond laser processing
Source: Nanophotonics. 2025 Mar 18;14(5):625–34. doi: 10.1515/nanoph-2024-0763 (PMC11953717; doi:10.1515/nanoph-2024-0763)
Supplement: Supplementary file 1 — Supplementary Material Details [file j_nanoph-2024-0763_suppl_001.docx]

**Optical transparent metamaterial emitter with multi-band compatible camouflage based on femtosecond laser processing: supplemental document**

Shu-Wen Zheng^1^, Xiu-Yu Chen^1^, Jin-Long Huang^1^, Kun Yu^1^, Meng-Dan Qian *^1^ and Yu-Fang Liu *^1,2^

1 Hennan Key Laboratory of Infrared Spectrum Measures and Applications, School of Physics, Henan Normal University, Xinxiang, Henan 453007, China

2 Institute of Physics, Henan Academy of Sciences, Zhengzhou, 450046, China

* qianmengdan@htu.edu.cn, liuyufang2005@126.com.

The supplementary information includes：

S1. Computed absorptance spectra for different Ag grating diameters

S2. Additional information for the theoretical calculation of resonance

S3. Fabrication of one-dimensional grating

S4. SEM images of samples under different laser pulse widths

S5. The radiative temperature calculation

S6. The radiation temperature at different temperatures

S7. The average emissivity of the samples

S8. Optically transparent multiband compatible camouflage comparison

**S1. Computed absorptance spectra for different Ag grating diameters**


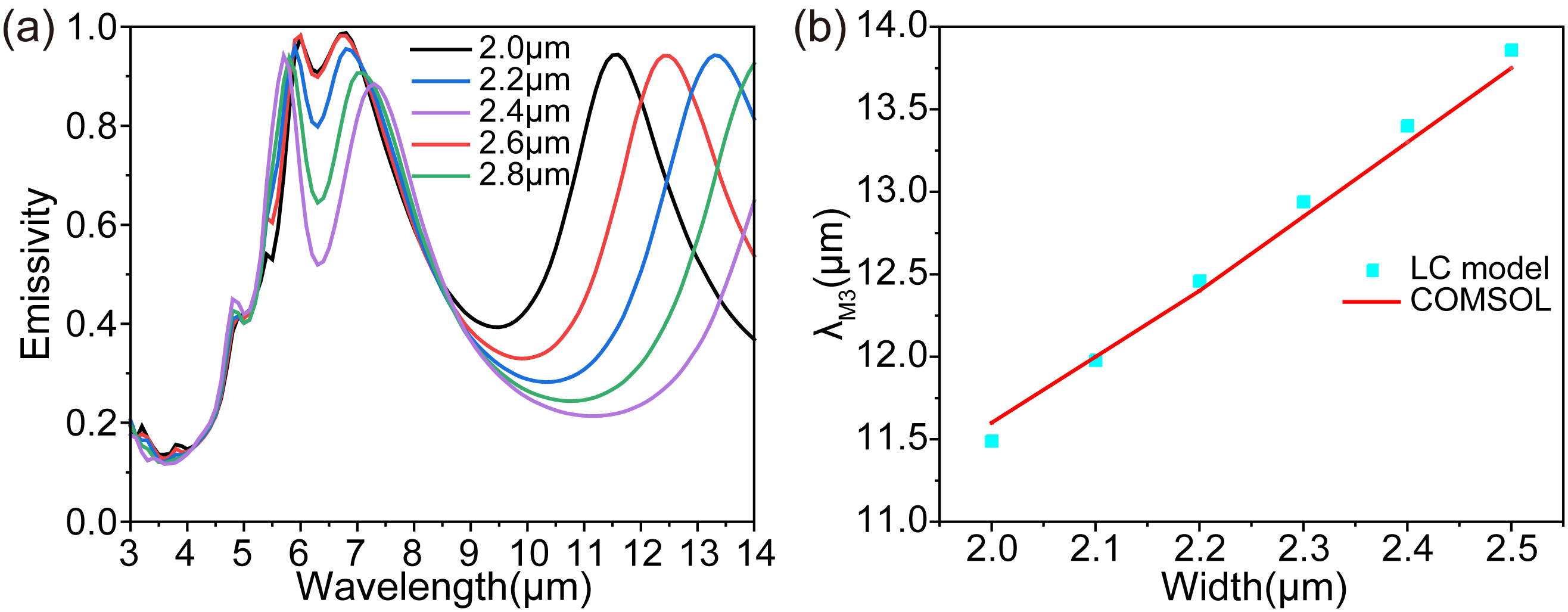


**Fig. S1.** (a) Computed absorptance spectra for different Ag grating diameters and equal unit cell pitch P=5.4μm. (b) Comparison between the magnetic polariton resonance wavelength λ_M3_ obtained from the computed spectra in (a) and λ_M3_ predicted with the LC model.

**S2. Additional information for the theoretical calculation of resonance**

To demonstrate that these resonances correspond to surface plasmon polariton (SPP) excitations, high-order magnetic resonances, and anti-reflection resonances coupling, we first calculated the SPP resonances for a 2D periodic array of Ag grating on an ITO substrate using the implicit relation[1]:

$\lambda_{SPP}=\frac{P}{\sqrt{j^{2}+k^{2}}}Re\left( \frac{\epsilon_{m}\left( \lambda\right)\epsilon_{d}}{\epsilon_{m}\left( \lambda\right){+\epsilon}_{d}} \right)$ (S1)

Here, P= 4 µm is the period of the 2D grating, (j; k) = {(±1; 0), (±1; ±1), (±2; 0), ...} represents the mode order index for the corresponding SPP resonance and Re(·) denotes the real part operator. The complex dielectric function of Ag, ϵm(λ), was obtained from literature, while ϵd represents the dielectric function of air. Our calculations revealed that $\lambda_{SPP}$ ∼ 5.4 µm for the mode order (±1, 0) and shifts to shorter wavelengths for higher diffraction orders. Secondly, the magnetic field distributions for these resonant modes, M1 and M2, is illustrated in Figs. 3(a) and 3(b), respectively.  The magnetic field distribution at the M1 and M2 are not only confined to the intermediate ZnS film between the Ag grating and bottom layers (indicative of a typical third-order magnetic resonance), but also in the interface between the ZnS film and the bottom ITO film (known as anti-reflection resonance). Specifically, for M1, the magnetic field intensity between the ITO film and Ag grating is greater than that of M2, whereas the magnetic field intensity at the ZnS-bottom ITO film interface is smaller. Conversely, for M1, the magnetic field intensity at the ZnS-bottom ITO film interface is greater than that of M2, whereas the magnetic field intensity between the ITO film and Ag grating is smaller. With a grating width of 3µm, period of 5.4µm, and dielectric layer thickness of 650nm, the wavelength of high-order magnetic resonance is 5.5µm, and the wavelength of anti-reflection resonance is 7.6µm. In M1, the influence of higher-order magnetic resonance is more significant, while in M2, the influence of anti-reflection resonance is greater. Hence, similar magnetic field distributions between M1 and M2, certain differences are observed. Consequently, the peaks M1 and M2 correspond to a mixed mode of coupling between SPP excitations, higher-order magnetic resonance, and anti-reflection resonance.

**S3. Fabrication of one-dimensional grating**

We manufactured the ITO-ZnS-Ag one-dimensional grating structure by femtosecond laser direct writing technique. The thickness of the dielectric layer ZnS was 450 nm, the thickness of the metal layer Ag and the grating was 18 nm, the width of the grating was 3 µm, and the structure period was 5 µm. We implemented the basic manufacturing test and manufactured the 15 × 15 mm sample. From Fig. S3 (a), it can be observed that the absorption spectrum displays two distinct absorption peaks at wavelengths of 5.1 μm and 7.4 μm. The emissivity of the emitter was measured in the actual thermal environment spanning 100-250 ℃, and the results presented in Fig. S3 (b). The sample effectively maintain its spectral selectivity well all over the measured temperature range. However, the optical performance of the resulting one-dimensional grating structure was found to be unsatisfactory. Specifically, the average emissivity in the non-atmospheric window range of 5-8 μm was found to be low, which compromised its performance in certain thermal management applications. Additionally, the thermal stability of the one-dimensional grating structure was observed to be suboptimal. As shown in the Fig. S3(c), the spectral emissivity of ITO-ZnS-Ag 2D grating structure. The experimentally measured spectrum of the sample showed that femtosecond laser direct writing has the ability to process MDM structures, which provides a new idea for IR camouflage structure manufacturing.


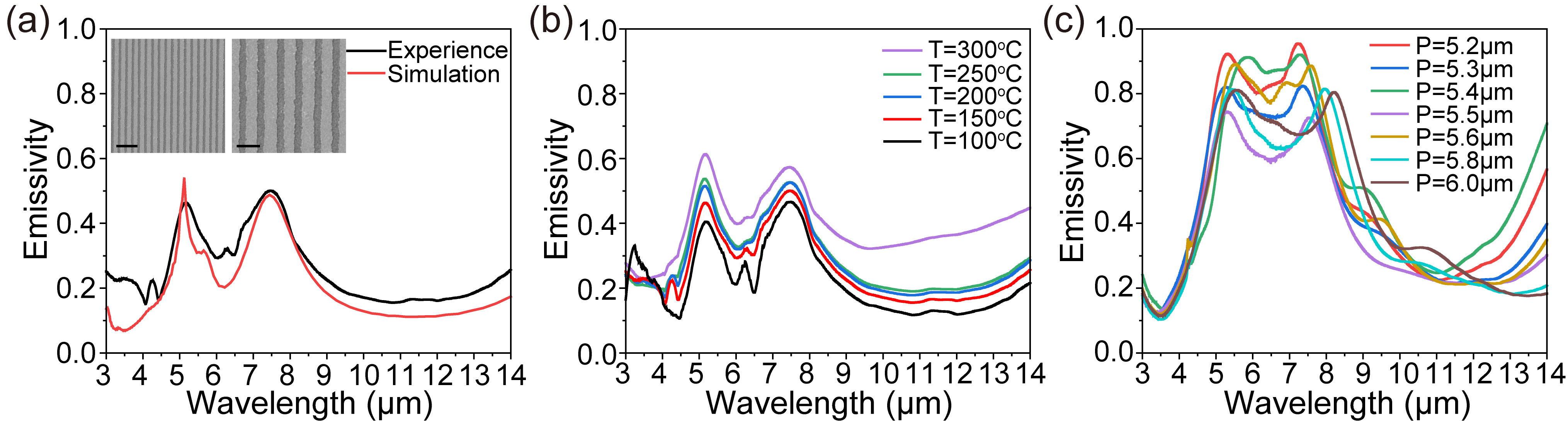


**Fig. S2.** (a) The simulated and experimentally emissivity of the designed structures in 3-14µm and the SEM images of the sample. Scale bar: 10 μm (top) and 1 μm (bottom), respectively. (b) The spectral emissivity of the emitter is measured across different temperatures utilizing Fourier Transform Infrared (FTIR) spectroscopy. (c) The spectral emissivity of ITO-ZnS-Ag 2D grating structure by femtosecond laser direct writing technique across different pitch.

**S4. SEM images of samples under different laser pulse widths**


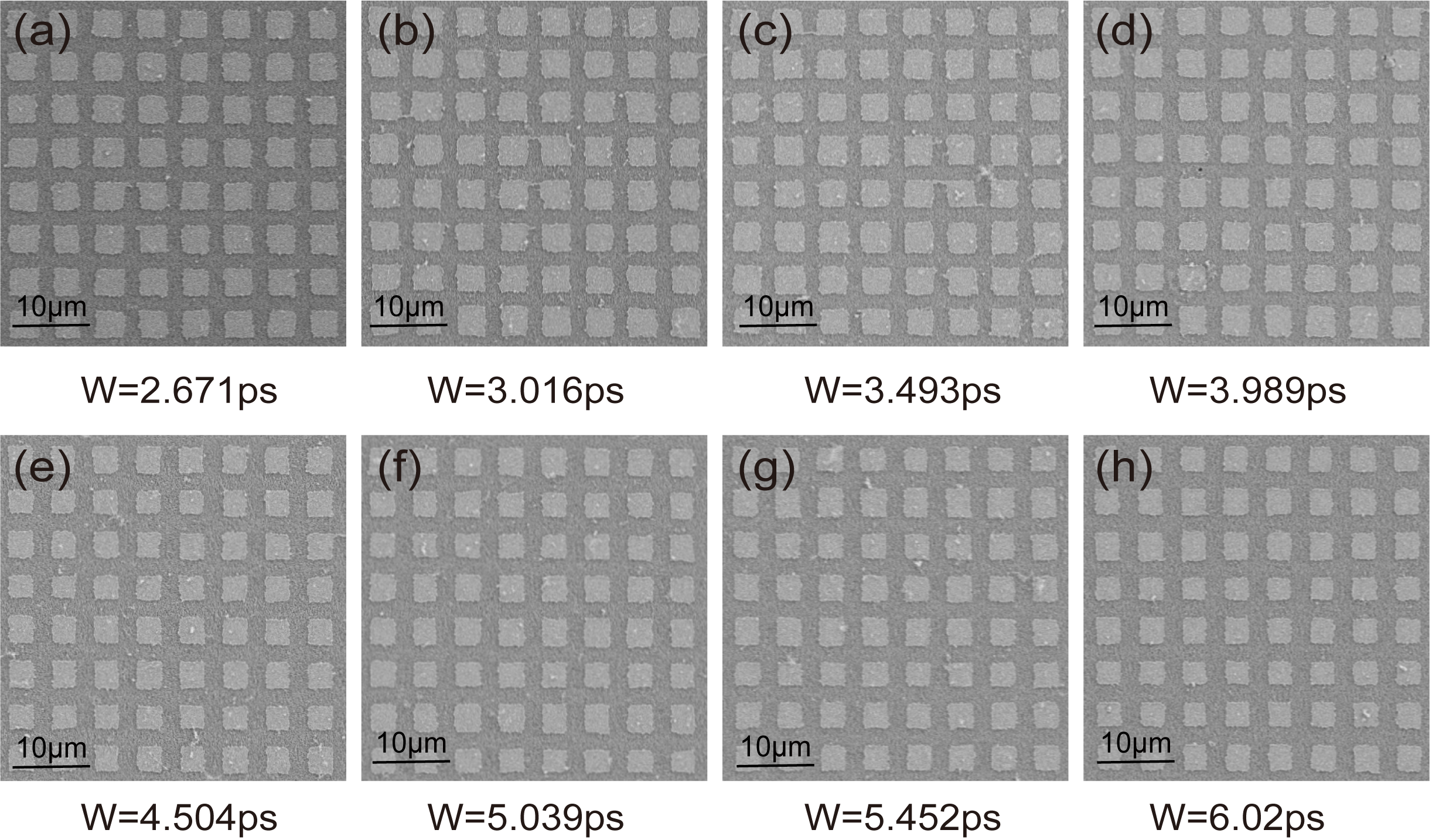


**Fig. S3.** (a–h) SEM images of metasurface samples under the influence of different laser pulse widths.

**S5. The radiative temperature calculation**

In practical outdoor scenarios, the reflection of sunlight must be taken into account during the design of IR camouflage, as the peak irradiance of sunlight within the IR band can be comparable to that of a 300 °C blackbody. To accurately simulate the power distribution captured by an infrared camera from an object, it is imperative to consider both the radiation intensity emitted by the object itself and the reflection of ambient radiation, including sunlight, by the object's surface[2]:

$P\left( \varepsilon,T \right)=P_{rad}\left( \varepsilon,T \right)+P_{ref}\left( \varepsilon,\varepsilon_{a},T_{a} \right)$ (S2)

$P_{rad}\left( \varepsilon,T \right)=C\int_{\lambda_{1}}^{\lambda_{2}} \varepsilon\left( \lambda\right)\cdot\frac{hc^{2}}{\lambda^{5}}\left( e^{\frac{hc}{\lambda k_{B}T}-1} \right)^{-1}d\lambda$ (S3)

$P_{ref}\left( \varepsilon,T \right)=C\int_{\lambda_{1}}^{\lambda_{2}} \left[ 1-\varepsilon\left( \lambda\right) \right]\varepsilon_{a}\left( \lambda\right)\cdot\frac{hc^{2}}{\lambda^{5}}\left( e^{\frac{hc}{\lambda k_{B}T_{a}}-1} \right)^{-1}d\lambda$ (S4)

Where, P(ε, T) denotes the total power detected from the object's surface, which comprises the radiation intensity emitted by the object (P_rad_) and the reflection of ambient radiation by the object (P_ref_). Here, ε(λ) and ε_a_(λ) represent the spectral emissivity of the object and the ambient spectral emissivity, respectively.  *T* stands for the object's temperature, while *T_a_*_​_ signifies the ambient temperature. [λ1, λ2] delineates the operational spectrum of the IR camera, and *C* represents the angular integration constant.


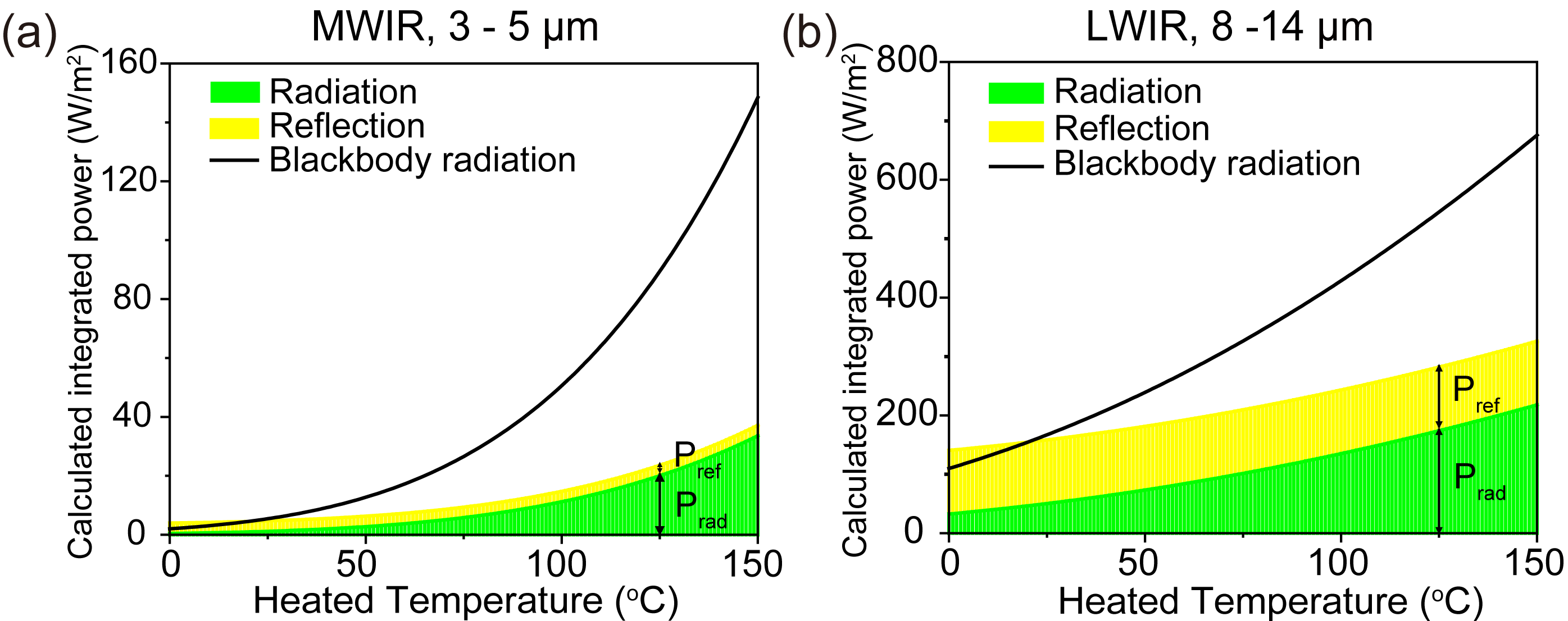


**Fig. S4.** Depicts the correlation between the sample's temperature and the total power captured by the IR camera. This power emanates from two sources: the sample's radiation (Prad, denoted by the green area) and the reflection of the ambient radiation by the sample (Pref, represented by the yellow area). The black line illustrates the integrated power of a blackbody and the ambient temperature is maintained at 20°C. (a) 3-5µm. (b) 8-14µm.

**S6. The radiation temperature at different temperatures**

**
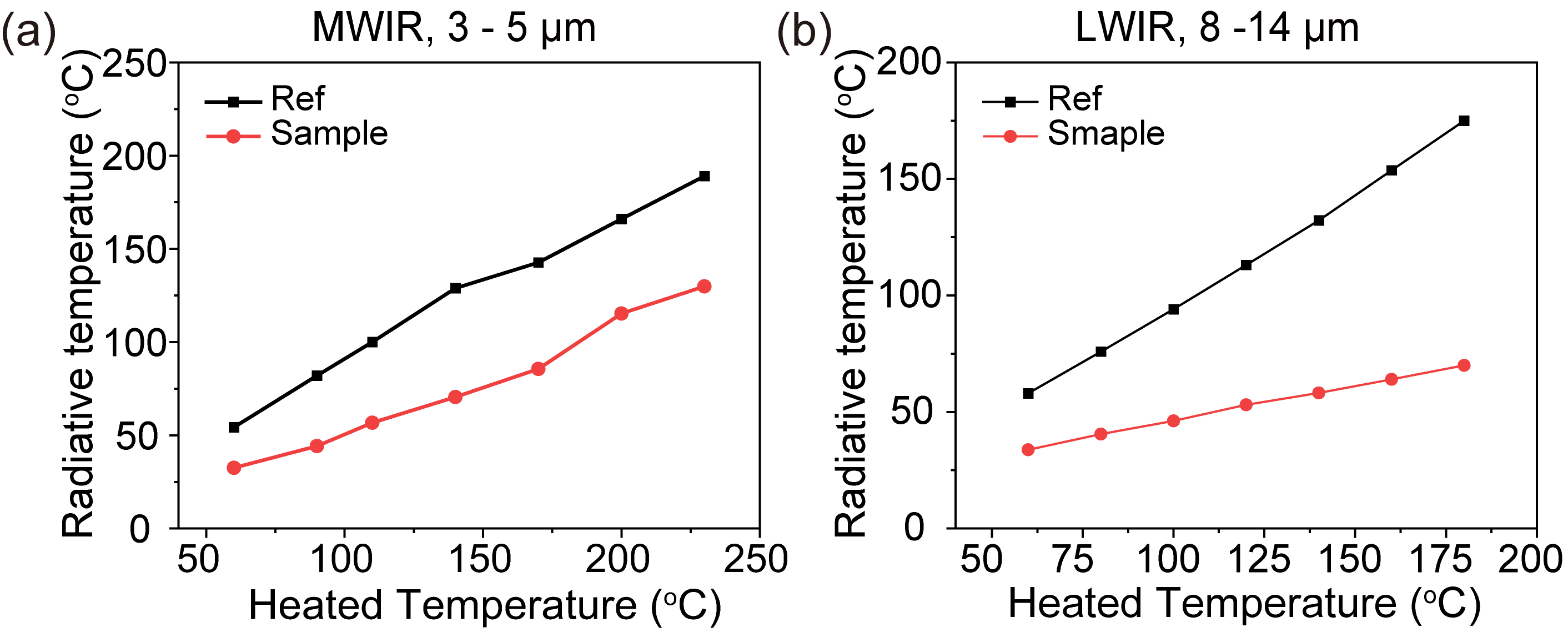
**

**Fig. S5.** The variation in radiation temperature across various heating temperatures. (a) 3-5µm. (b) 8-14µm.

**S7. The average emissivity of the samples**

**
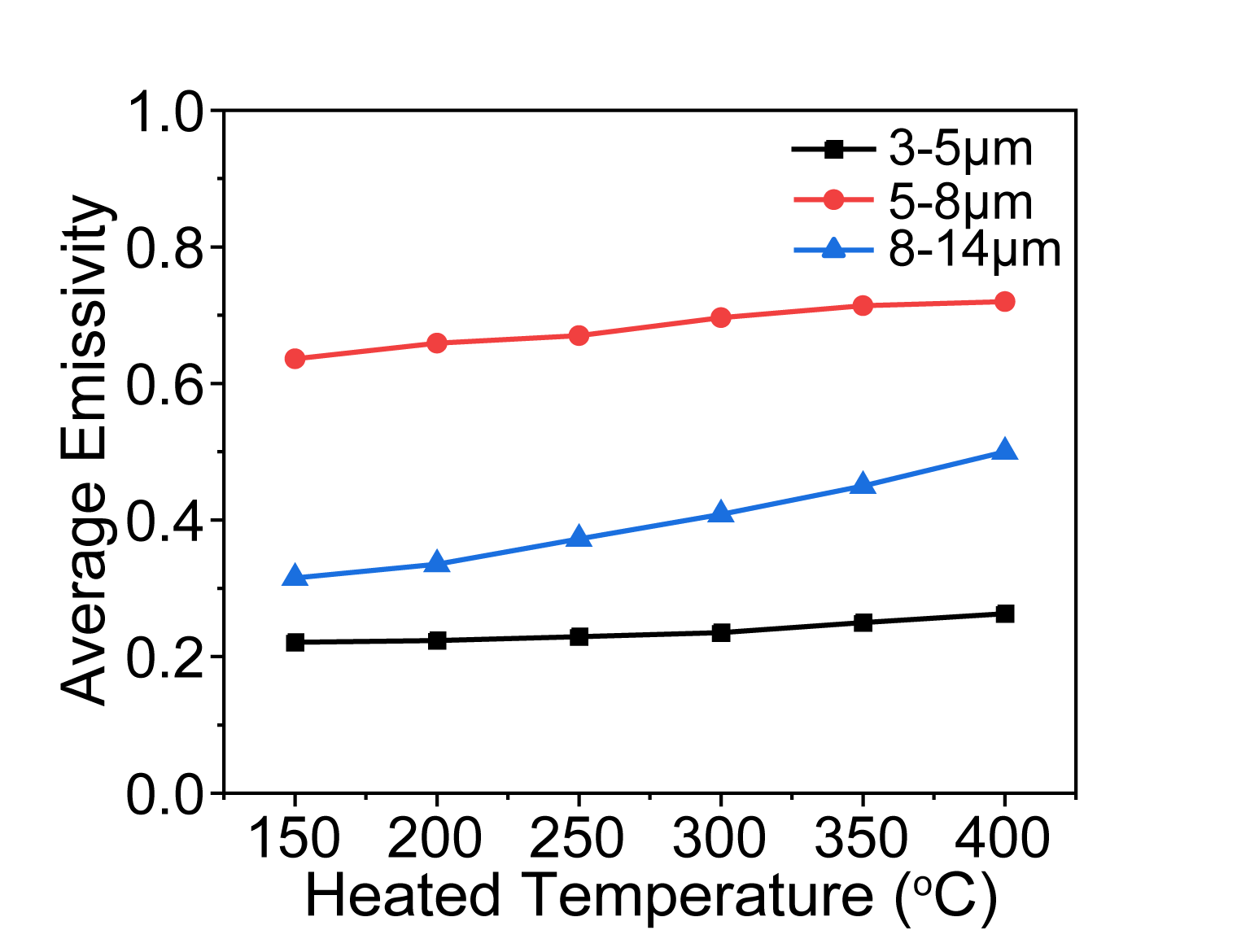
**

**Fig. S6.** The average emissivity of the samples in different bands in the temperature range of 150-400 ℃.

**S8. Optically transparent multiband compatible camouflage comparison**

Table S1. Optically transparent multiband compatible camouflage performance comparison

| Reference | Material | Type | Visible performance | NIR laser R_1.06µm_ | NIR laser R_1.55µm_ | Thermal camouflage | Thermal management | Experiment |
| --- | --- | --- | --- | --- | --- | --- | --- | --- |
| [3] | Ag/GST/TiO_2_/Ge/ZnS | Film | _ | 0.01 | _ | R_3-5µm_：0.78  R_8-14µm_：0.84 | A_5-8µm_=0.65 | No |
| [4] | ITO/FR4/Gu/Si/Ge/ZnS | Film | _ | _ | 0.3 | R_3-5µm_：0.89  R_8-14µm_：0.88 | A_5-8µm_=0.61 | Yes |
| [5] | ITO/Si_3_N_4_/Au | Film and grating | 0.44 | _ | _ | R_3-5µm_：0.64  R_8-14µm_：0.75 | A_5-8µm_=0.52 | Yes |
| [6] | Ag/ZnO | Film | 0.63 | _ | _ | R_3-5µm_：0.76  R_8-14µm_：0.91 | A_5-8µm_=0.57 | Yes |
| [7] | Ag/Si_3_N_4_ | Film and grating | 0.7 | _ | 0.01 | R_3-5µm_：0.86  R_8-14µm_：0.92 | A_5-8µm_=0.61 | No |
| Our work | ITO/ZnS/Ag | Film and grating | 0.36 | 0.3 | 0.1 | R_3-5µm_：0.69  R_8-14µm_：0.70 | A_5-8µm_=0.64 | Yes |

Compared with previous research, our work introduces a multi-band compatible camouflage scheme with effective thermal management. Specifically, most existing research focuses on achieving either visible-band stealth compatible with the infrared range or infrared stealth compatible with certain laser bands. In contrast, our work offers a sufficiently wide range of stealth coverage, including the visible, near-infrared (including both 1.06µm and 1.55µm), and infrared bands. Furthermore, the structure has been experimentally demonstrated excellent stealth performance, especially in the near-infrared and mid-infrared bands, with particularly outstanding thermal management capabilities. The data have been added to the supporting information and. The revised content is shown as follows.

References

[1] J. C. Tong, L. Y. M. Tobing, Y. Luo, D. W. Zhang, and D. H. Zhang, “Single Plasmonic Structure Enhanced Dual-band Room Temperature Infrared Photodetection,” *Scientific Reports,* vol. 8, pp. 9, 2018.

[2] M. Y. Pan, Y. Huang, Q. Li, H. Luo, H. Z. Zhu, S. Kaur *et al.*, “Multi-band middle-infrared-compatible camouflage with thermal management via simple photonic structures,” *Nano Energy,* vol. 69, pp. 9, 2020.

[3] Z. Y. Chen, X. H. Wu, Q. L. Kang, H. T. Liu, K. Yu, and K. H. Zhang, “Visible color camouflage and infrared, laser band stealth, compatible with dual-band radiative heat dissipation,” *International Communications in Heat and Mass Transfer,* vol. 159, pp. 8, 2024.

[4] H. Z. Zhu, Q. Li, C. N. Tao, Y. Hong, Z. Q. Xu, W. D. Shen *et al.*, “Multispectral camouflage for infrared, visible, lasers and microwave with radiative cooling,” *Nature Communications,* vol. 12, no. 1, pp. 8, 2021.

[5] N. Lee, J. S. Lim, I. Chang, D. Lee, and H. H. Cho, “Transparent Metamaterials for Multispectral Camouflage with Thermal Management,” *International Journal of Heat and Mass Transfer,* vol. 173, pp. 8, 2021.

[6] Y. J. Wu, J. Luo, M. B. Pu, B. Liu, J. J. Jin, X. Li *et al.*, “Optically transparent infrared selective emitter for visible-infrared compatible camouflage,” *Optics Express,* vol. 30, no. 10, pp. 17259-17269, 2022.

[7] J. Nong, X. P. Jiang, X. L. Wei, Y. Y. Zhang, N. Li, X. Li *et al.*, “Optical transparent metamaterial with multi-band compatible camouflage based on inverse design,” *Optics Express,* vol. 31, no. 20, pp. 33622-33637, 2023.
